# Anti-EGFR monoclonal antibody plus chemotherapy for treating advanced non-small cell lung cancer: A meta-analysis

**DOI:** 10.1097/MD.0000000000027954

**Published:** 2021-11-24

**Authors:** Wenqing Luo, Yuanqi Li, Fei Ye, Qiangming Li, Guoqing Zhang, Jindong Li, Xiangnan Li

**Affiliations:** a Department of thoracic surgery, First Affiliated Hospital of Zhengzhou University, Zhengzhou, Henan Province, China; b XiangYa School of Public Health, Central South University, Changsha, Hunan Province, China.

**Keywords:** anti-epidermal growth factor receptor mAb, chemotherapy, combination therapy, meta-analysis, non-small-cell lung cancer

## Abstract

**Background:**

The use of standard cytotoxic chemotherapy seems to have reached a “treatment plateau”. The application of anti-epidermal growth factor receptor (EGFR) monoclonal antibodies (mAbs) is a new strategy for non-small-cell lung cancer (NSCLC) therapy. We aimed to comprehensively assess the efficacy and safety of anti-EGFR-mAbs plus chemotherapy as first-line therapy for advanced NSCLC.

**Methods:**

According to inclusion and exclusion criteria, we conducted a comprehensive literature search of electronic databases. From the included trials, information on overall survival (OS), progression-free survival (PFS), objective response rate (ORR), and adverse events (AEs) was extracted.

**Results:**

The research showed that compared with chemotherapy alone, anti-EGFR-mAb plus chemotherapy combinations significantly improved OS (HR = 0.88, 95%CI: 0.83-0.94, *P* < .0001), PFS (HR = 0.89, 95%CI: 0.83-0.95, P = 0.0004) and ORR (OR = 1.39, 95%CI: 1.13-1.69, *P* = .001). Meta subgroup analyses manifested that the OS of patients with squamous NSCLC treated with anti-EGFR-mAb plus chemotherapy combinations was notably better than that of patients with non-squamous NSCLC treated with the same combinations (HR = 0.82, 95%CI: 0.73-0.92, *P* = .0005). Compared with the chemotherapy group, combination of chemotherapy and anti-EGFR mAb showed increase in incidences of severe AEs (> = grade 3) that mainly include, leukopenia (OR = 1.53, 95%CI: 1.28-1.82, *P* < .00001), febrile neutropenia (OR = 1.35, 95%CI: 1.06-1.71, *P* = .02), hypomagnesemia (OR = 5.68, 95%CI: 3.54-9.10, *P* < .00001), acneiform rash (OR = 35.88, 95%CI: 17.37-74.10, *P* < .00001), fatigue (OR = 1.24, 95%CI: 1.02-1.49, *P* = .03), diarrhea (OR = 1.69, 95%CI: 1.16-2.47, *P* = .006), and infusion-related reactions (OR = 3.78, 95%CI: 1.93-7.41, *P* = .0001).

**Conclusion:**

Adding an anti-EGFR-mAb to the standard platinum-based chemotherapy regimens used for the first-line treatment of advanced NSCLC resulted in statistically notable improvements in OS, PFS, and ORR. In particular, anti-EGFR-mAb and chemotherapy combinations achieved greater survival benefits in patients with squamous NSCLC than in those with non-squamous NSCLC. In addition, the safety profile of chemotherapy plus anti-EGFR-mAb combinations was acceptable compared to that of chemotherapy alone.

## 1 Introduction

Worldwide, lung cancer is the most common malignancy, and it remains the leading cause of cancer incidence and mortality.^[1]^ Small-cell lung cancer and non-small-cell lung cancer (NSCLC) are the two main types of lung cancer, accounting for approximately 15% and 85% of cases, respectively.^[2]^ NSCLC lacks typical symptoms in its early stages, and therefore, almost 70% of NSCLC cases have spread to localized or distant parts of the body at the time of diagnosis; such cases are classified as advanced NSCLC (stages IIIB-IV).^[3]^ The standard chemotherapy regimen for advanced NSCLC is platinum plus a second drug, usually paclitaxel, gemcitabine, vincristine, docetaxel or pemetrexed.^[4]^ Nevertheless, the response rate of this dual chemotherapy regimen is only about 20%, and the median overall survival (OS) is only 8 to 10 months.^[5]^ A sequence of randomized trials has indicated that different platinum-based dual chemotherapy regimens have similar efficacy as first-line therapy for NSCLC.^[6–10]^ Furthermore, adding a cytotoxic drug to the combination of chemotherapeutic drugs has been proven to increase drug toxicity without benefiting OS.^[9,11,12]^ The prognosis of advanced NSCLC is poor, and the classic cytotoxic chemotherapy regimen appears to have reached a “therapeutic plateau”. Accordingly, we need to develop new treatments and drugs to improve the survival outcomes of patients with NSCLC.

About 80% of patients with NSCLC overexpress epidermal growth factor receptor (EGFR).^[13]^ Its overexpression has an important effect on tumor cell growth, proliferation, metastasis, and angiogenesis and is closely related to the prognosis and survival rate of resected tumor patients. Therefore, EGFR-targeted therapy has become a new strategy for NSCLC therapy.^[14]^ Currently, cetuximab and necitumumab are the two most commonly used anti-EGFR monoclonal antibodies (mAbs). Cetuximab is a human-mouse chimeric IgG1 anti-EGFR-mAb that selectively binds to EGFR, competitively inhibits its binding to endogenous ligands and inhibits the activation of epidermal growth factor (EGF) and downstream intracellular signal transduction.^[15]^ The FLEX trial^[16]^ showed that adding an anti-EGFR-mAb, cetuximab, to vincristine and cisplatin chemotherapy in the first-line treatment of patients with NSCLC expressing EGFR remarkably improved OS (median, 11.3 months vs 10.1 months, HR = 0.871, 95%CI: 0.762-0.996, *P* = .044). Nonetheless, in another randomized trial, Lynch,^[17]^ similar OS benefits were achieved by a cetuximab plus paclitaxel (paclitaxel or docetaxel) and carboplatin combination and chemotherapy alone (median, 9.69 months vs 8.38 months, HR = 0.890, 95%CI: 0.754-1.051, *P* = .169). The second generation of recombinant human IgG1 anti-EGFR-mAb, necitumumab, which binds to EGFR and competitively inhibits ligand binding, thus downstream signal transduction and blocking receptor activation.^[18]^ The SQUIRE trial^[19]^ indicated that adding necitumumab to chemotherapy could notably improve the OS of patients with squamous NSCLC (median, 11.5 months vs 9.9 months, HR = 0.84, 95%CI: 0.74-0.96, *P* = .01). In contrast, the INSPIRE trial^[20]^ revealed that adding necitumumab to chemotherapy did not improve OS in patients with non-squamous NSCLC (median, 11.3 months vs 11.5 months, HR = 1.01, 95%CI: 0.84-1.21, *P* = .96). Additionally, 2 meta-analyses that considered the same four trials to appraise the efficacy of cetuximab plus chemotherapy reached different conclusions: one showed significantly improved progression-free survival (PFS), while the other did not.^[3,21]^ Another meta-analysis to determine the efficacy of chemotherapy combined with cetuximab showed that cetuximab plus chemotherapy had more benefits for NSCLC than chemotherapy alone.^[22]^ Moreover, a meta-analysis showed that the OS of necitumumab plus chemotherapy was significantly better than that of chemotherapy alone, but there were no remarkable differences in objective response rate (ORR) and PFS.^[23]^ The limitations of these meta-analyses include that the trials included only one anti-EGFR-mAb, the sample sizes of patients were too small, and analysis of publication bias was lacking.

Were these contradictory results caused by clinical or methodological heterogeneity, or were they purely accidental? To better explain the available evidence and to overcome the limitations of these studies, we conducted the meta-analysis. We aimed to comprehensively determine the efficacy and safety of chemotherapy plus an anti-EGFR-mAb in the first-line therapy of advanced NSCLC.

## 2 Methods

### 2.1 Ethics statement

All analyses in this article were based on previously published studies, so ethical approval and patient consent are not applicable.

### 2.2 Literature search

The two authors (Luo and Zhang) independently performed a comprehensive review of the literature in the following electronic databases (without any date or language restrictions): 1). Cochrane Central Register of Controlled Trials (CENTRAL, from inception to 10 October 2020); 2). MEDLINE (access through PubMed (1966 to 10 October 2020)); 3). Web of Science (1950 to 10 October 2020); 4). Embase (1980 to 10 October 2020); 5). ClinicalTrials.gov (from inception to 10 October 2020); and 6). WHO International Clinical Trials Registry Platform (from inception to 10 October 2020)

The keywords of the literature search included “chemotherapy,” “cetuximab,” “necitumumab” “NSCLC,” and “non-small-cell lung cancer”. Additionally, we retrieved the reference list of relevant articles, including reviews.

### 2.3 Study inclusion and exclusion criteria

The 2 authors (Luo and Zhang) simultaneously and independently scanned the titles and abstracts of the papers. The authors examined the full texts of the papers that seemed to meet the predetermined inclusion and exclusion criteria. The authors discussed and resolved the differences through negotiation.

The inclusion criteria were as follows: phase II or III randomized controlled trials (RCTs) designed for patients with stage IIIB or IV NSCLC; studies in which simultaneous or sequential radiation therapy was not permitted; studies in which participants were randomly allocated to the anti-EGFR-mAb (cetuximab or necitumumab) plus standard chemotherapy arm as the experimental group or to the chemotherapy alone arm as the control group; and studies in which survival outcomes and the incidence of adverse events (AEs) were reported or in which there were sufficient data available to calculate these results.

The exclusion criteria were as follows: single-arm clinical trials and studies without data on survival outcomes and AEs; observational studies; systematic reviews; meta-analyses.

### 2.4 Quality and risk of bias assessment for the included studies

We used Cochrane Collaboration tools, which assesses random sequence generation, allocation concealment, blinding of participants and personnel, blinding of outcome assessment, incomplete outcome data, selective outcome reporting and other biases, to evaluate the risk of deviations in randomized trials.^[24]^

### 2.5 Data extraction

The major outcome was OS. The subordinate outcomes were PFS, ORR, and AEs.

### 2.6 Statistical analysis

For survival outcomes, we typically calculated the logarithmic HR and standard error (SE), and the research results were pooled by the inverse variance method of survival outcomes and presented in the form of combined HRs and 95% CIs. For dichotomous data such as ORR and AEs, we generally used the Mantel-Haenszel method to estimate the merged HRs and 95% CIs. The Mantel-Haenszel fixed-effects method and random-effects method were used in the meta-analysis. A *P* value was considered to indicate significance at the level of < .05. The heterogeneity between the included studies was tested by the chi-square test; *P* > .1 meant there was no heterogeneity, and *P* < .1 indicated that there was heterogeneity. In addition, the I^2^ test was used to evaluate the heterogeneity: I^2^ < 25% = low heterogeneity; 25% < I^2^ < 50% = moderate heterogeneity; and I^2^ > 50% = high heterogeneity. When I^2^ > 50% or *P* < .1, the random effects model was used; otherwise, the fixed-effects model was used. To identify and determine the source of the heterogeneity, we performed a subgroup analysis based on the treatment regimen and disease characteristics, represented as the following different groups: 1). squamous NSCLC and non-squamous NSCLC; 2). cetuximab plus chemotherapy and necitumumab plus chemotherapy; and 3). different chemotherapy regimens plus an anti-EGFR-mAb. We used Egger test to check the funnel chart to determine whether there was publication bias in the included literature. When P value at the level of > 0.05, there was no publication bias; conversely, *P* value at the level of ≤ .05, there was publication bias.

We used forest plots to present the pooled HR for survival outcomes (OS, PFS) and OR for dichotomous data with corresponding 95% CI. All statistical analyses were performed using Review Manager 5 software (RevMan version 5.4) and R 4.0.3 software.

## 3 Results

### 3.1 Literature search results

**Figure 1:**
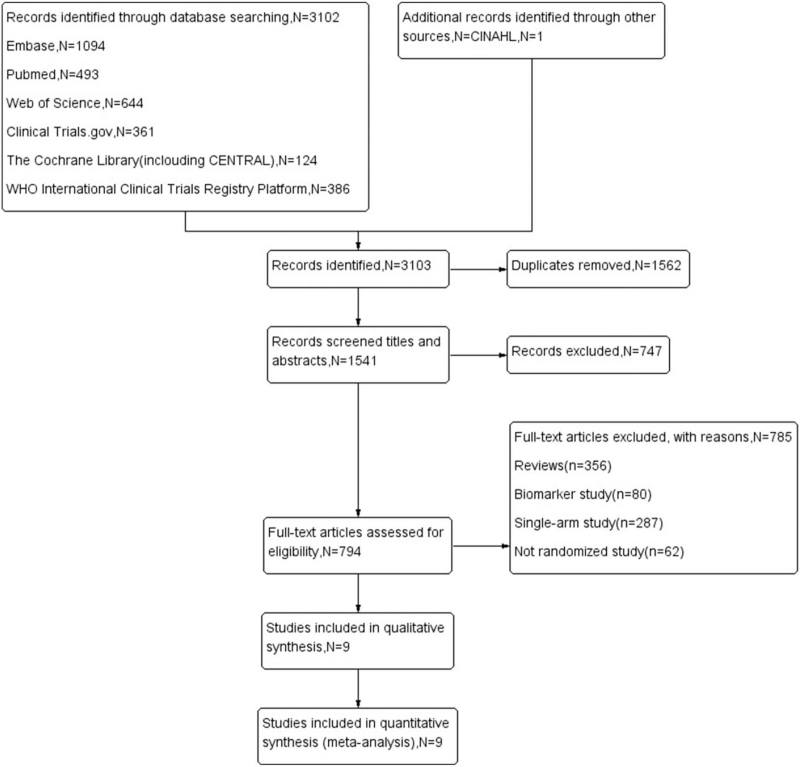
Flow chart showing study selection.

This meta-analysis retrieved 9 studies that conformed to the inclusion and exclusion criteria. The details of the literature retrieval process and the reasons for study exclusion are shown in the flow chart in Figure 1. Table 1 shows the characteristics of the included studies.

**Table 1:** Characteristics of included randomized clinical trials.

		Number of	Number of	Median Age,		
Study	Study Arms	Patients	Males	Years	Inclusion Period	Phase
R. Rosell (2008)	Cetuximab + NP	43	33	58	February 2002 to May 2003	II
	NP	43	31	57		
Thomas J. Lynch (2010)	Cetuximab + TP	338	192	64	January 2005 to November 2006	III
	TP	338	204	65		
Robert Pirker (2009)	Cetuximab + NP	557	385	59	October 2004 to January 2006	III
	NP	568	405	60		
Charles A. Butts (2007)	Cetuximab + GP	65	25	66	January 2005 to September 2005	II
	GP	66	33	64		
Roy S Herbst (2018)	Cetuximab + TP + (-)Bevacizumab	656	385	63	August 2009 to May 2014	III
	TP + (-)Bevacizumab	657	359	63		
Nick Thatcher (2015)	Necitumumab + GP	545	450	62	January 2010 to February 2012	III
	GP	548	458	62		
Luis Paz-Ares (2015)	Necitumumab + AP	315	214	61	November 2009 to February 2011	III
	AP	318	210	60		
David R. Spigel (2017)	Necitumumab + TP	110	87	66	February 2013 to April 2015	II
	TP	57	44	65		
Satoshi Watanabe (2019)	Necitumumab + GP	90	79	67	May 2013 to June 2017	II
	GP	91	81	65		

AP = Pemetrexed + Cisplatin, GP = Gemcitabine + Cisplatin, NP = Vinorelbine + Cisplatin, TP = Paclitaxel + Carboplatin.

### 3.2 Risk of bias

A summary of the bias risk analysis is shown in Supplemental Digital Content 1.

### 3.3 Efficacy

#### 3.3.1 OS and PFS

**Figure 2:**
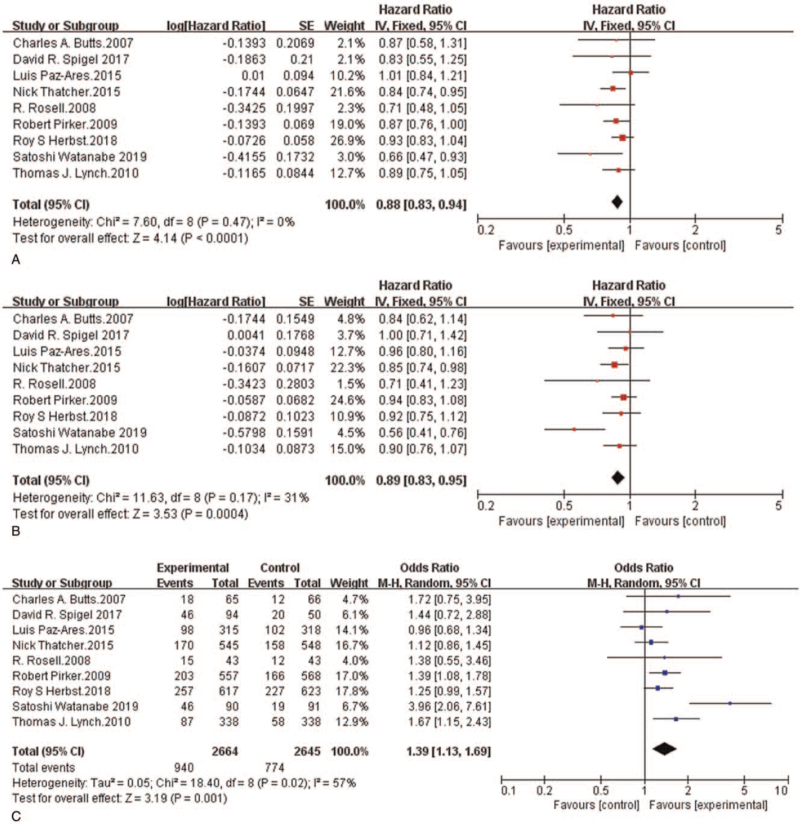
Forest plots for overall survival pooled hazard ratio (A), progression-free survival pooled hazard ratio (B) and objective response rate pooled odds ratio (C): Chemotherapy plus anti-EGFR-mAb versus chemotherapy alone for advanced NSCLC. NSCLC = non-small-cell lung cancer; anti-EGFR-mAb = anti-epidermal growth factor receptor monoclonal antibody; CI = confidence interval; SE = standard error; Ch^2^ = chi-squared test; df = degree of freedom; I^2^ = I-squared.

The meta-analysis showed that in the therapy of patients with NSCLC, the anti-EGFR-mAb and standard first-line chemotherapy combination provided a remarkable benefit in terms of efficacy outcomes compared to chemotherapy alone (Fig. 2A–C). The median OS for the chemotherapy and anti-EGFR-mAb was from 8.3 months^[25]^ to 14.9 months,^[26]^ and that for chemotherapy alone was from 7.3 months^[25]^ to 11.5 months.^[20]^ Eight of the nine included studies showed that compared with chemotherapy alone, chemotherapy plus anti-EGFR-mAb had a longer median OS. Only one study manifested that the median OS with necitumumab plus chemotherapy was slightly shorter than that with chemotherapy alone (11.3 vs 11.5).^[20]^ Compared with chemotherapy alone, randomized chemotherapy plus anti-EGFR-mAb combinations had a notably lower risk of death (hazard ratio (HR) = 0.88, 95% confidence interval (CI): 0.83-0.94, *P* < .0001) (Fig. 2A).

The median PFS of patients treated with chemotherapy plus an anti-EGFR-mAb was from 4.2 months^[26]^ to 5.7 months,^[19]^ and that of patients treated with chemotherapy alone was from 4 months^[26]^ to 5.6 months.^[18,20]^ The meta-analysis indicated that patients who received randomized chemotherapy plus an anti-EGFR-mAb had a markedly lower risk of disease progression than those who received chemotherapy alone (HR = 0.89, 95%CI: 0.83-0.95, *P* = .0004) (Fig. 2B).

#### 3.3.2 ORR

The ORR of the chemotherapy plus anti-EGFR-mAb combination ranged from 25.7%^[17]^ to 51.1%,^[26]^ and that of chemotherapy alone ranged from 17.2%^[17]^ to 40%.^[18]^ The meta-analysis showed that the ORR of patients who received randomized chemotherapy plus an anti-EGFR-mAb was obviously higher than that of patients who received chemotherapy alone (OR = 1.39, 95%CI: 1.13-1.69, *P* = .001) (Fig. 2C).

### 3.4 Heterogeneity analysis of efficacy

Our study demonstrated that all the included studies had low statistical heterogeneity for OS (I^2^ = 0%, *P* = .47) and PFS (I^2^ = 31%, P = 0.17)] but high heterogeneity for ORR (I^2^ = 57%, *P* = .02). To determine the source of ORR heterogeneity, we used the literature exclusion method to analyze ORR heterogeneity. The analysis showed that the “Satoshi Watanabe 2019” study may have been the source of heterogeneity. The results before and after the ORR sensitivity analysis were inconsistent, indicating that the combined ORR results are not robust and need to be interpreted with caution (See Table, Supplemental Digital Content 2 which shows the sensitivity analysis for objective response rate).

### 3.5 Subgroup analyses of efficacy

**Figure 3:**
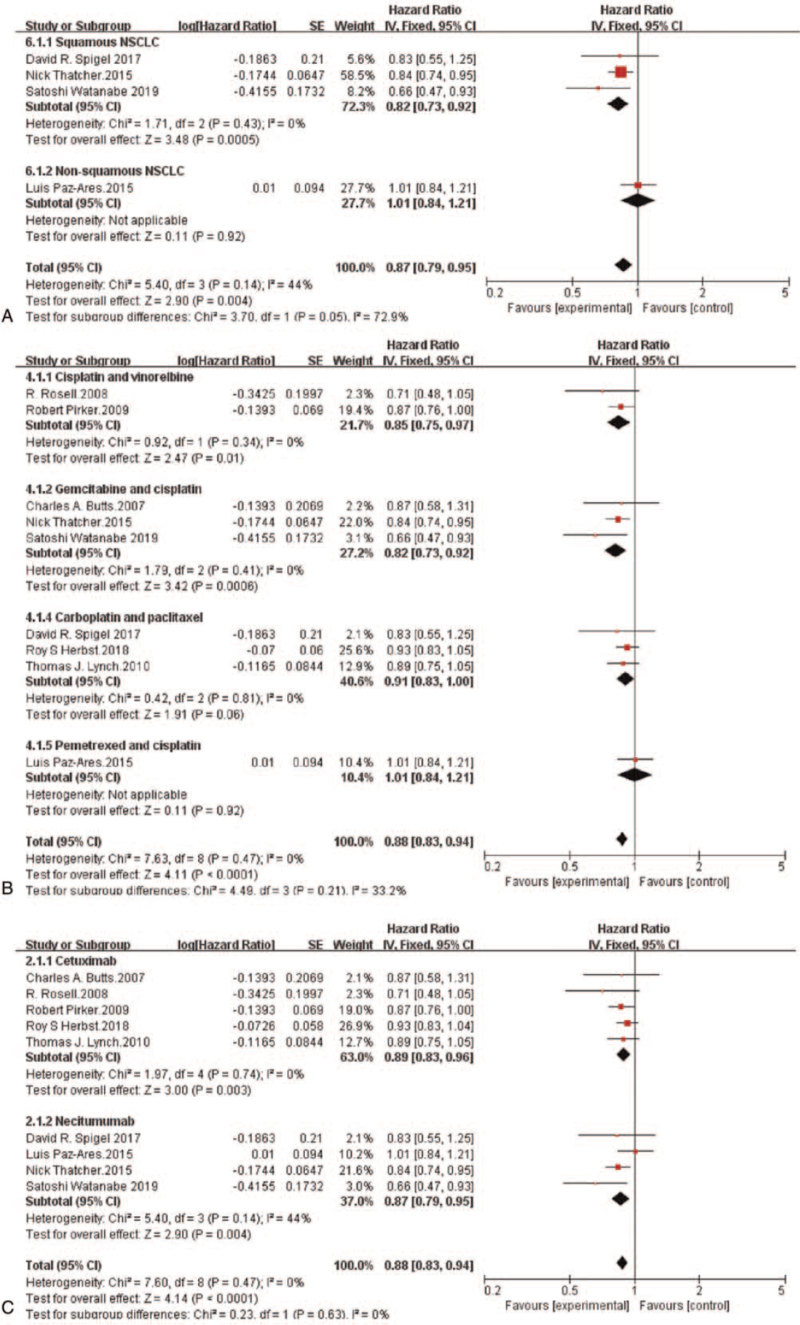
Forest plots for overall survival pooled hazard ratio by histology (A), chemotherapy (B) and anti-EGFR-mAb (C): Chemotherapy plus anti-EGFR-mAb versus chemotherapy alone for advanced NSCLC. NSCLC = non-small-cell lung cancer; anti-EGFR-mAb = anti-epidermal growth factor receptor monoclonal antibody; CI = confidence interval; SE = standard error; Ch^2^ = chi-squared test; df = degree of freedom; I^2^ = I-squared.

Subgroup analysis showed that in terms of OS, patients with squamous NSCLC who received randomized chemotherapy plus an anti-EGFR-mAb had a notably lower risk of death than those who received chemotherapy alone (HR = 0.82, 95%CI: 0.73-0.92, *P* = .0005). However, randomized chemotherapy plus an anti-EGFR-mAb did not reduce the risk of death in patients with non-squamous NSCLC compared with chemotherapy alone (HR = 1.01, 95%CI: 0.84-1.21, *P* = .92) (Fig. 3A). Moreover, an OS benefit was found in the anti-EGFR-mAb plus gemcitabine and cisplatin subgroup (HR = 0.82, 95%CI: 0.73-0.92, *P* = .0006) and in the anti-EGFR-mAb plus vinorelbine and cisplatin subgroup (HR = 0.85, 95%CI: 0.75-0.97, *P* = .01) (Fig. 3B). The risk of death in patients with NSCLC was obviously reduced in both the cetuximab plus chemotherapy subgroup (HR = 0.89, 95%CI: 0.83-0.96, *P* = .003) and the necitumumab plus chemotherapy subgroup (HR = 0.87, 95%CI: 0.79-0.95, *P* = .004) (Fig. 3C).

**Figure 4:**
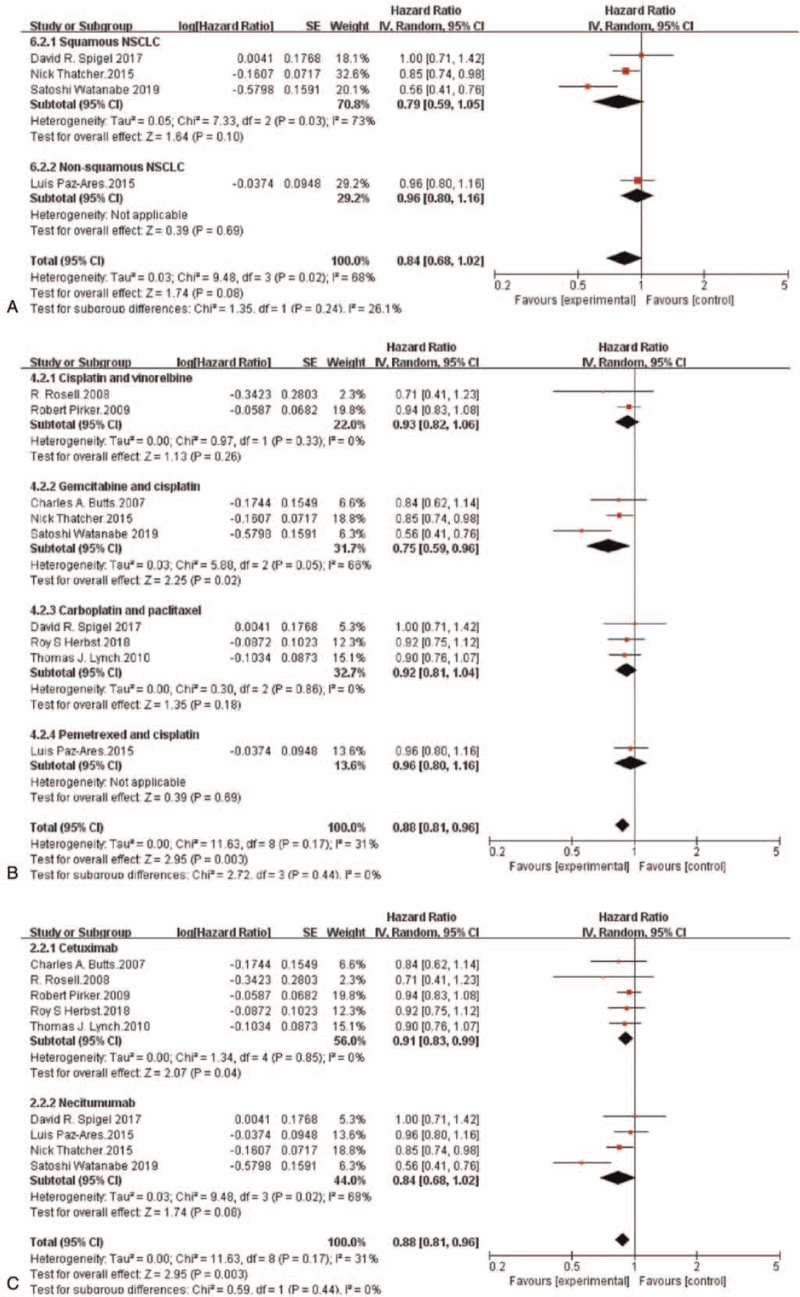
Forest plots for progression-free survival pooled hazard ratio by histology (A), chemotherapy (B) and anti-EGFR-mAb (C): Chemotherapy plus anti-EGFR-mAb versus chemotherapy alone for advanced NSCLC. NSCLC = non-small-cell lung cancer; anti-EGFR-mAb = anti-epidermal growth factor receptor monoclonal antibody; CI = confidence interval; SE = standard error; Ch^2^ = chi-squared test; df = degree of freedom; I^2^ = I-squared.

Subgroup analysis demonstrated that no statistically notable improvement in PFS was provided by adding an anti-EGFR-mAb to chemotherapy in patients with non-squamous NSCLC (HR = 0.96, 95%CI: 0.80-1.16, *P* = .69) or squamous NSCLC (HR = 0.79, 95%CI: 0.59-1.05, *P* = .10) (Fig. 4A). Furthermore, a PFS benefit from adding an anti-EGFR-mAb was observed only in the gemcitabine plus cisplatin subgroup (HR = 0.75, 95%CI: 0.59-0.96, *P* = .02) (Fig. 4B). The risk of disease progression in patients with NSCLC was markedly reduced in the cetuximab plus chemotherapy subgroup (HR = 0.91, 95%CI: 0.83-0.99, *P* = .04). However, the PFS in the necitumumab plus chemotherapy subgroup was not statistically improved (HR = 0.84, 95%CI: 0.68-1.02, *P* = .08) (Fig. 4C).

**Figure 5:**
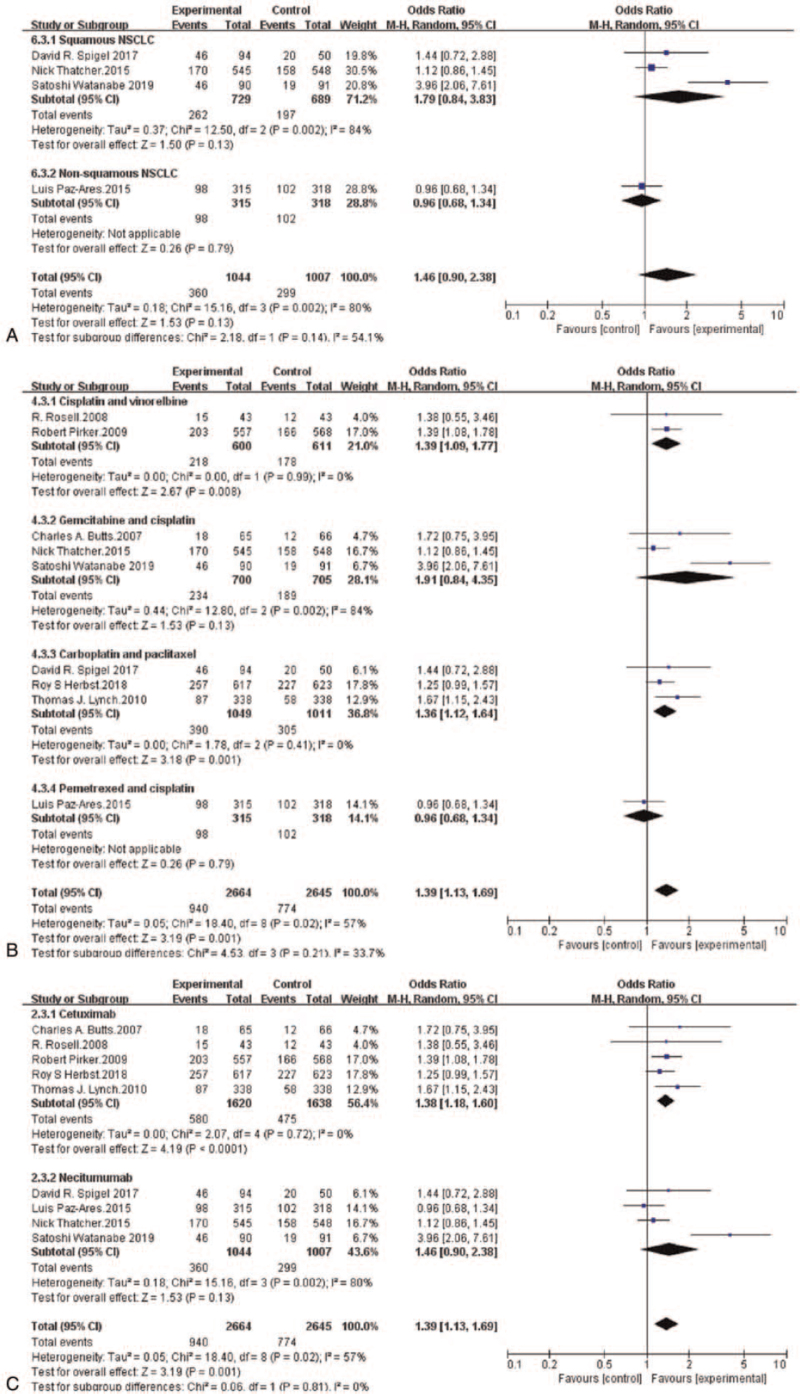
Forest plots for objective response rate pooled odds ratio by histology (A), chemotherapy (B) and anti-EGFR-mAb (C): Chemotherapy plus anti-EGFR-mAb versus chemotherapy alone for advanced NSCLC. NSCLC = non-small-cell lung cancer; anti-EGFR-mAb = anti-epidermal growth factor receptor monoclonal antibody; CI = confidence interval; SE = standard error; Ch^2^ = chi-squared test; df = degree of freedom; I^2^ = I-squared.

Subgroup analysis revealed that there no remarkable difference in ORR was provided by adding an anti-EGFR-mAb to chemotherapy in patients with squamous NSCLC or non-squamous NSCLC (Fig. 5A). Additionally, an ORR benefit from adding an anti-EGFR-mAb was found in the vinorelbine plus cisplatin subgroup (HR = 1.39, 95%CI: 1.09-1.77, *P* = 0.008) and carboplatin plus paclitaxel subgroup (HR = 1.36, 95%CI: 1.12-1.64, *P* = .001) (Fig. 5B). Patients with NSCLC in the cetuximab plus chemotherapy subgroup had an obviously improved ORR (HR = 1.38, 95%CI: 1.18-1.60, *P* < 0.0001). Nevertheless, the ORR in the necitumumab plus chemotherapy subgroup was not significantly increased (HR = 1.46, 95%CI: 0.90-2.38, *P* = .13) (Fig. 5C).

### 3.6 Safety

According to the WHO criteria for toxicity and side effects of chemotherapeutic drugs, the most common grade 3 and 4 AEs included leukopenia, thrombocytopenia, neutropenia, fatigue, vomiting, diarrhea, rash, and so on. Our research found that the incidences of leukopenia, febrile neutropenia, hypomagnesemia, acneiform rash, fatigue, diarrhea, and infusion-related reactions in grade 3 and 4 AEs were observably higher in the experimental group than in the control group (See Figure, Supplemental Digital Content 3–15 which shows the forest plot of AEs in this study). The detailed analysis is shown in Table 2.

**Table 2:** Pooled OR and 95% CI for Adverse Events by Preferred Terms and Composite Categories.

Adverse Events by Preferred Terms	Chemotherapy	Chemotherapy	Odds Ratio,	Heterogeneity		
and Composite Categories	Plus EGFR mAbs,Event/Total	Alone, Event/Total	95%CI	*P* value	I^2^	*P* value
Haematological adverse events						
Leukopenia	402/1542	296/1557	1.53 (1.28–1.82)	.83	0%	< .00001
Neutropenia	990/2644	906/2622	1.16 (0.94–1.44)	.01	58%	.16
Febrile neutropenia	174/2144	134/2110	1.35 (1.06–1.71)	.55	0%	.02
Thrombocytopenia	181/1792	176//1748	1.01 (0.80–1.27)	.67	0%	.93
Anemia	226/2017	244/1990	0.90 (0.74–1.09)	.75	0%	.27
Hypomagnesemia	113/1729	20/1697	5.68 (3.54–9.10)	.67	0%	<.00001
Non-haematological adverse events						
Acneiform rash	246/2644	5/2622	35.88 (17.37–74.10)	.71	0%	< .00001
Fatigue	264/2512	215/2488	1.24 (1.02–1.49)	.35	11%	.03
Nausea	26/431	19/429	1.38 (0.76–2.51)	.36	2%	.3
Vomiting	43/654	45/671	0.98 (0.63–1.51)	.57	0%	.92
Diarrhea	73/2102	44/2121	1.69 (1.16–2.47)	.59	0%	.006
Dyspnea	50/612	51/628	1.01 (0.67–1.51)	.17	46%	.98
Infusion-related Reactions	39/1475	10/1489	3.78 (1.93–7.41)	.83	0%	.0001

anti-EGFR-mAb = anti-epidermal growth factor receptor monoclonal antibody, CI = confidence interval, I^2^ = I-squared, OR = odds ratio.

### 3.7 Publication bias

To minimize the potential publication bias, we adopted scientific search methods and strict inclusion criteria. Moreover, according to the funnel chart used to evaluate publication bias and the Egger test, our main results found no significant asymmetry (See Figure, Supplemental Digital Content 16–21 which shows the publication bias for the efficacy outcomes).

## 4 Discussion

EGFR has been identified as a therapeutic target for NSCLC. First, anti-EGFR-mAb binds to EGFR and competitively inhibits its binding to endogenous ligands, blocking the transmission of crucial pathways and inhibiting the growth of tumor cells expressing EGFR.^[27]^ Second, some studies have shown that some anti-EGFR-mAbs can induce an immune response and enhance the cytotoxic response to chemotherapy.^[15,28,29]^ However, the advantages of combination therapy involving anti-EGFR mAb and platinum based chemotherapy have not been studied before. Our research showed that adding cetuximab or necitumumab to the standard platinum combination chemotherapy in advanced NSCLC could bring modest benefits to all efficacy endpoints (OS, PFS, and ORR). Our study included the two most commonly used anti-EGFR-mAbs, cetuximab and necitumumab, and relevant high-quality RCTs to further explore the efficacy and safety of anti-EGFR-mAbs plus standard chemotherapy regimens. Yang et al found that the OS and ORR of the chemotherapy plus cetuximab group were markedly better than those of the chemotherapy alone group, but the difference in PFS was not remarkable.^[3]^ In another meta-analysis, Ilic^[23]^ found that the OS of the chemotherapy plus necitumumab group was notably better than that of the chemotherapy alone group, but there were no notable differences in PFS or ORR, which is consistent with the results of our subgroup analysis. Consequently, the combination of an anti-EGFR-mAb with a platinum-based dual chemotherapy regimen is a reasonable strategy for the clinical treatment of advanced NSCLC.

Among the three trials enrolling patients with squamous NSCLC, adding necitumumab to chemotherapy achieved OS benefits.^[18,19,26]^ In only the INSPIRE trial that examined patients with non-squamous NSCLC, adding necitumumab to cisplatin and pemetrexed did not benefit OS, PFS, or ORR.^[20]^ Furthermore, given the increased number of grade 3 and higher AEs, necitumumab was not considered a good option for non-squamous NSCLC. Similarly, another subgroup meta-analysis revealed that the OS of patients with squamous cell cancer seemed to benefit most from cetuximab (HR = 0.77, 95%CI: 0.64-0.93, *P* = .01).^[21]^ Although these trials focused on different anti-EGFR-mAbs, we must consider that the benefits of cetuximab and necitumumab in the squamous cell cancer subgroup may not have been found by accident. This possibility needs to be further tested and has two possible explanations. First, it has been proven that EGFR expression is higher in squamous cell cancer than in non-squamous cell cancer.^[30–32]^ In addition, a study further analyzing data collected from the FLEX trial showed that only high EGFR expression (IHC score > 200; score range 0–300) could predict a survival benefit from adding cetuximab to chemotherapy, and high EGFR expression proved to be an effective tumor biomarker.^[33]^ Another study based on the SQUIRE trial revealed that the benefit of adding necitumumab to chemotherapy was significantly increased for the EGFR-expressing subgroup of patients with squamous NSCLC.^[34]^ This finding may be related to the remarkably improved OS seen in squamous cell cancer patients treated with chemotherapy plus cetuximab or necitumumab, which is considerably better than the OS seen in non-squamous cell cancer patients treated with the same regimen. Second, anti-EGFR-mAbs induce antibody-dependent cell-mediated cytotoxicity (ADCC) against EGFR-expressing lung cancer cells. A study found that cetuximab has the potential to induce ADCC against EGFR-expressing lung cancer cells.^[15]^ Another study showed that necitumumab could inhibit EGFR-dependent tumor cell proliferation and exert cytotoxic effects on tumor cells via ADCC.^[28]^ Therefore, it is reasonable to assume that patients with squamous NSCLC may benefit more from anti-EGFR-mAb therapy than other NSCLC patients because these antibodies are known to induce ADCC. Another study showed that high expression of EGFR, with or without the presence of EGFR mutations, was a potentially useful tumor biomarker for predicting the survival benefit from first-line chemotherapy plus cetuximab in NSCLC.^[35]^ Given the value of tumor biomarkers for predicting the survival benefit for patients with NSCLC, we will continue to develop different tumor biomarkers for clinical applications.

The subgroup analysis of different EGFR-mAbs showed that patients with NSCLC in the cetuximab plus chemotherapy subgroup^[16,17,25,36,37]^ and the necitumumab plus chemotherapy subgroup^[18–20,26]^ had remarkably improved OS. Although cetuximab and necitumumab are different anti-EGFR-mAbs, their mechanisms of action are similar. Both of them are IgG1 anti-EGFR-mAbs that bind to EGFR with high affinity and competitively inhibit ligand binding, thus inhibiting the activation and downstream signal transduction of EGF.

The subgroup analysis according to chemotherapy regimen showed that only the gemcitabine and cisplatin doublet chemotherapy plus anti-EGFR-mAb subgroup achieved significant gains in OS and PFS. In contrast, the pemetrexed and cisplatin chemotherapy plus anti-EGFR-mAb subgroup failed to achieve benefits for OS, PFS, and ORR. One study showed that there was a synergistic anticancer effect between cetuximab and docetaxel, gemcitabine, and cisplatin, but pemetrexed did not produce such effects.^[38]^ Therefore, this anticancer synergy may be the reason why pemetrexed plus an anti-EGFR-mAb does not benefit OS, while gemcitabine plus an anti-EGFR-mAb does. However, such evidence is limited, and it is not clear which anti-EGFR-mAb plus chemotherapy regimen will induce the greatest benefit in patients with NSCLC. Further studies to determine the answer are warranted.

Our study showed that compared with the control group, the severe AEs (> = grade 3) notably increased incidences in the experimental group were leukopenia, febrile neutropenia, hypomagnesemia, acneiform rash, fatigue, diarrhea, and infusion-related reactions. These findings are basically consistent with the results of another meta-analysis.^[3]^ The toxicity characteristics of the anti-EGFR-mAb plus chemotherapy combinations were consistent with those previously reported, and there was no significant heterogeneity of these adverse events (*P* value > = 0.3). Overall, this research showed that the safety of chemotherapy plus anti-EGFR-mAb combinations was acceptable. For patients who are going to receive anti-EGFR-mAb plus chemotherapy regimens in the future, attention should be paid to the prevention and treatment of such AEs. Effective prevention and timely treatment can reduce the pain from treatment and improve the quality of life of patients.

Immune checkpoint inhibitors (ICIs) play a significant anti-tumor effect through T cell regulatory pathway, including immunotherapeutic drugs targeting programmed death receptor 1 (PD-1) / programmed death-ligand 1 (PD-L1), such as pembrolizumab, nivolumab, and atezolizumab. ICIs single drug or combination with chemotherapy has been recommended for second-line treatment of advanced NSCLC and first-line treatment of advanced NSCLC with positive PD-1/PD-L1 expression (≥50%) and no EGFR, ALK-driven gene mutations. Moreover, Sugiyama et al found that targeting EGFR in combination with anti-PD-1 mAb could increase the efficacy of lung cancer immunotherapy.^[39]^ EGFR-mAb as a single treatment of EGFR-activated mutant NSCLC or in combination with PD-1/PD-L1 inhibitors may be feasible for patients with EGFR-TKIs-resistant non-small cell lung cancer.

The low estimated risk of bias is an advantage of this meta-analysis. Moreover, our study included many RCTs, which resulted in a relatively large sample size. In addition, the included trials were stratified by histological type, which ensured balanced distributions in our subgroup analyses of squamous and non-squamous NSCLC cases. We also included trials that stratified patients according to the anti-EGFR-mAb and chemotherapy regimen used, which is an advantage of our study. We conducted publication bias analysis and sensitivity analysis of the included studies, which increased the credibility of the results.

Our research also has some limitations. Our study did not include trials of all anti-EGFR-mAbs, only the two most commonly used mAbs, cetuximab, and necitumumab. We were not able to access the original data for each experiment, and we could only analyze and group the available data. Some studies did not provide specific values for the outcomes of subgroup analysis, and we could not group patients according to sex, age, and other variables to perform additional subgroup analyses. Another major limitation is that we could not use EGFR expression as a biomarker for prognostic stratification because different evaluation criteria for EGFR expression were used in the studies.

## 5 Conclusion

This meta-analysis revealed that adding an anti-EGFR-mAb to the standard platinum chemotherapy for first-line treatment of advanced NSCLC obviously improved OS, PFS, and ORR outcomes. In particular, adding an anti-EGFR-mAb to chemotherapy achieved greater survival benefits in patients with advanced squamous NSCLC than in patients with non-squamous NSCLC. However, these results need to be interpreted cautiously because there was limited available data on patient characteristics and biomarkers, and these additional factors may be associated with the survival benefits derived from anti-EGFR-mAbs plus chemotherapy. The meta-analysis also showed that the safety of chemotherapy plus anti-EGFR-mAb combinations was acceptable compared with that of chemotherapy alone.

## Author contributions

GQZ, JDL and XNL conceived of the idea, designed the study. WQL searched the relevant database and wrote the manuscript. WQL, FY, YQL and QML analysed and interpreted the data. XNL and GQZ provided the examination for the methodology, reviewed and revised our manuscript. All authors read and approved the final manuscript.

**Conceptualization:** Guoqing Zhang.

**Data curation:** Fei Ye.

**Formal analysis:** Wenqing Luo.

**Funding acquisition:** Xiangnan Li.

**Investigation:** Yuanqi Li.

**Methodology:** Yuanqi Li.

**Resources:** Qiangming Li, Jindong Li.

**Supervision:** Guoqing Zhang, Jindong Li, Xiangnan Li.

**Validation:** Xiangnan Li.

**Visualization:** Wenqing Luo.

**Writing – original draft:** Wenqing Luo.

**Writing – review & editing:** Guoqing Zhang, Jindong Li, Xiangnan Li.

## Supplementary Material

Supplemental Digital Content

## Supplementary Material

Supplemental Digital Content

## Supplementary Material

Supplemental Digital Content

## Supplementary Material

Supplemental Digital Content

## Supplementary Material

Supplemental Digital Content

## Supplementary Material

Supplemental Digital Content

## Supplementary Material

Supplemental Digital Content

## Supplementary Material

Supplemental Digital Content

## Supplementary Material

Supplemental Digital Content

## Supplementary Material

Supplemental Digital Content
