# Supplementary material for: Anti-EGFR monoclonal antibody plus chemotherapy for treating advanced non-small cell lung cancer: A meta-analysis
Source: Medicine (Baltimore). 2021 Nov 24;100(47):e27954. doi: 10.1097/MD.0000000000027954 (PMC8615333; doi:10.1097/MD.0000000000027954)
Supplement: Supplemental Digital Content [file medi-100-e27954-s002.doc]

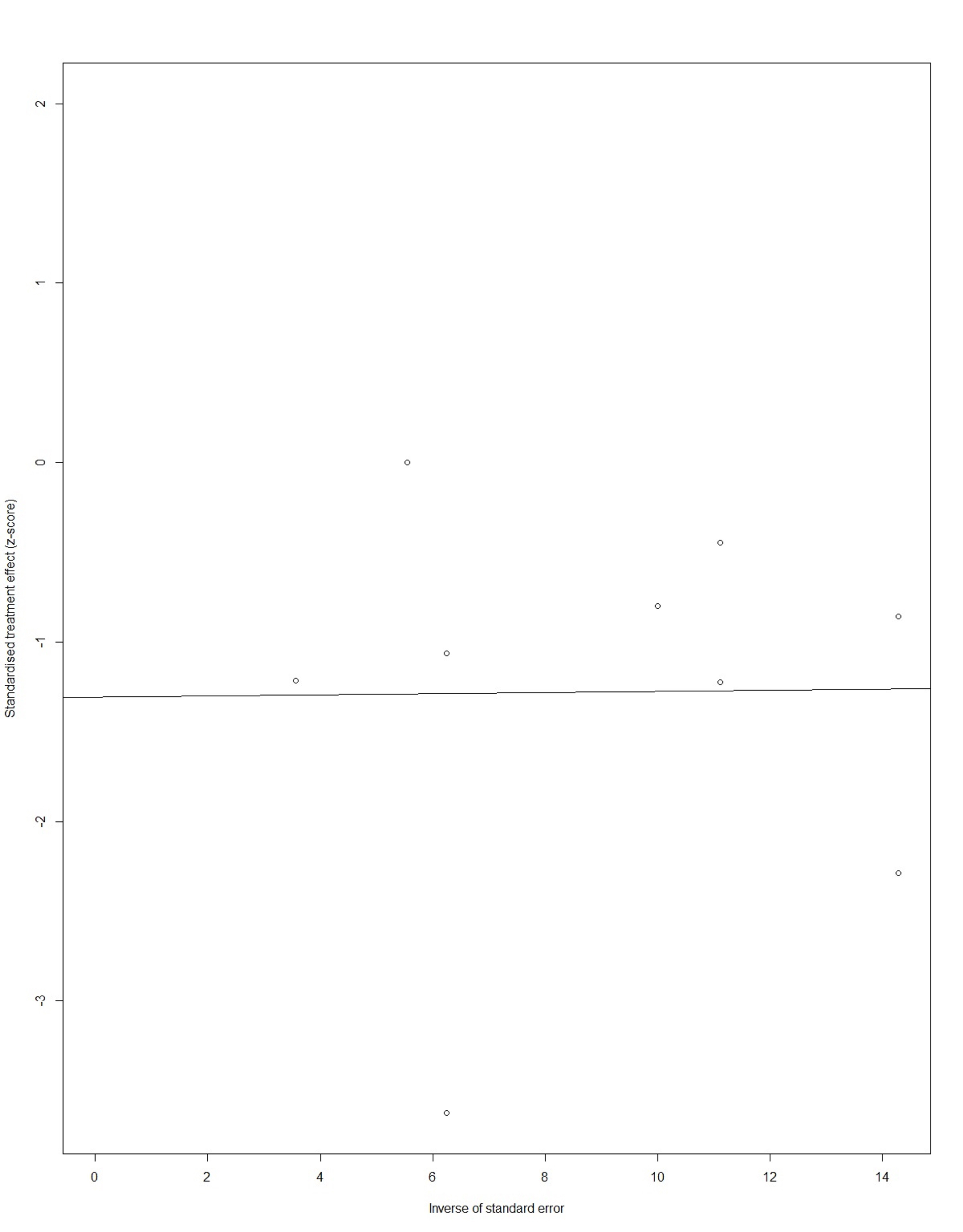


Supplemental Digital Content 19. Regression test for funnel plot of progression-free survival asymmetry. Chemotherapy plus anti-EGFR-mAb versus chemotherapy alone for advanced NSCLC. anti-EGFR-mAb= anti-epidermal growth factor receptor monoclonal antibody; NSCLC=non-small-cell lung cancer.


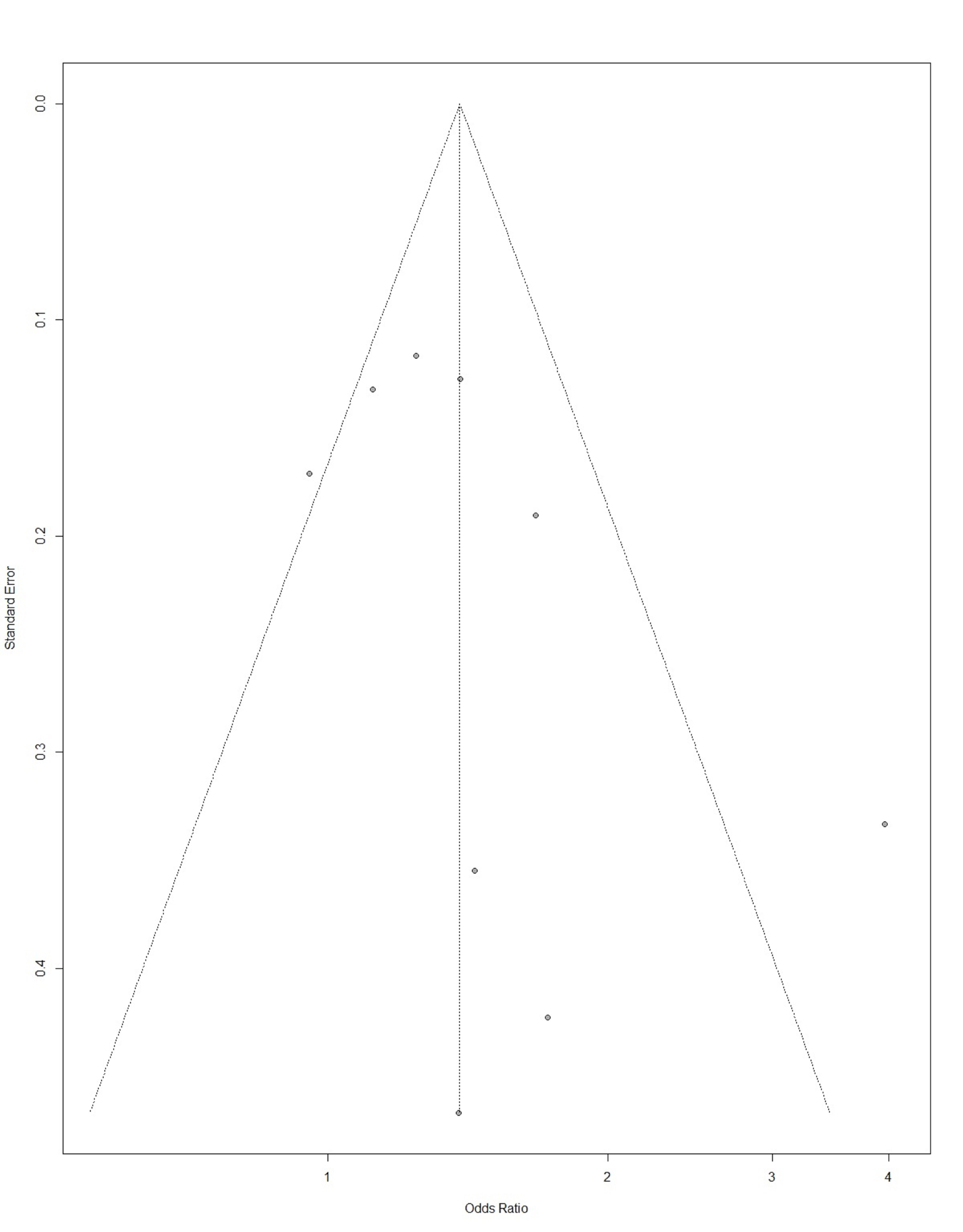


Supplemental Digital Content 20. Funnel plot for objective response rate. Chemotherapy plus anti-EGFR-mAb versus chemotherapy alone for advanced NSCLC. anti-EGFR-mAb= anti-epidermal growth factor receptor monoclonal antibody; NSCLC=non-small-cell lung cancer.


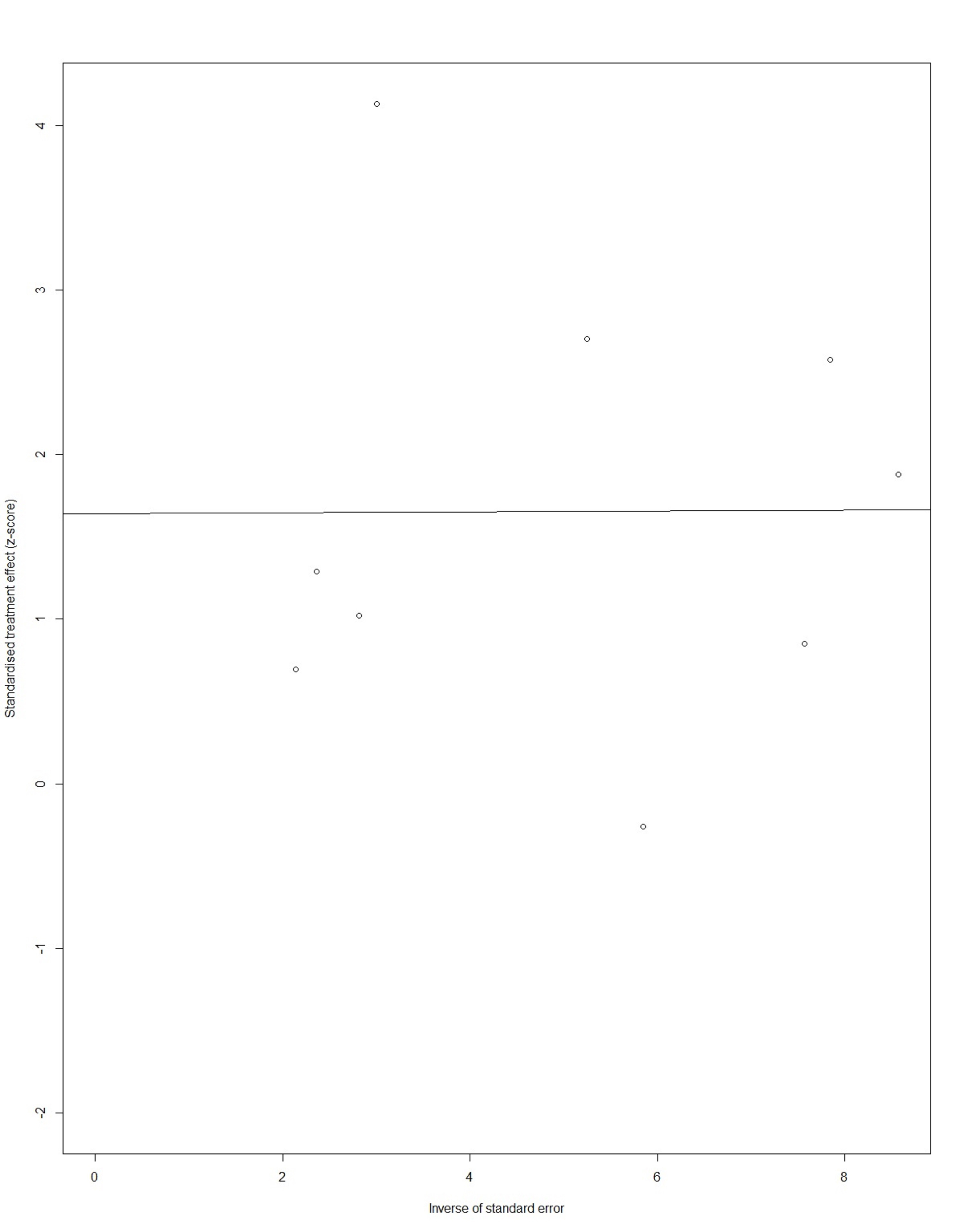


Supplemental Digital Content 21. Regression test for funnel plot of objective response rate asymmetry. Chemotherapy plus anti-EGFR-mAb versus chemotherapy alone for advanced NSCLC. anti-EGFR-mAb= anti-epidermal growth factor receptor monoclonal antibody; NSCLC=non-small-cell lung cancer.
