# Supplementary material for: Anti-EGFR monoclonal antibody plus chemotherapy for treating advanced non-small cell lung cancer: A meta-analysis
Source: Medicine (Baltimore). 2021 Nov 24;100(47):e27954. doi: 10.1097/MD.0000000000027954 (PMC8615333; doi:10.1097/MD.0000000000027954)
Supplement: Supplemental Digital Content [file medi-100-e27954-s003.doc]

Supplemental Digital Content 2. Sensitivity analysis for objective response rate. Chemotherapy plus anti-EGFR-mAb versus chemotherapy alone for advanced NSCLC. anti-EGFR-mAb= anti-epidermal growth factor receptor monoclonal antibody; NSCLC=non-small-cell lung cancer; CI=confidence interval; I2=I-squared; OR=odds ratio.

| **Study Excluded** | **Random Effect** | | **I^2^ (%)** | **I^2^-P Value** |
| --- | --- | --- | --- | --- |
|  | **OR** | **95%CI** |  |  |
| R. Rosell.2008 | 1.39 | 1.13-1.72 | 62 | 0.01 |
| Thomas J. Lynch.2010 | 1.35 | 1.09-1.68 | 58 | 0.02 |
| Robert Pirker.2009 | 1.41 | 1.10-1.80 | 61 | 0.01 |
| Charles A. Butts.2007 | 1.38 | 1.11-1.70 | 61 | 0.01 |
| Roy S Herbst.2018 | 1.44 | 1.12-1.85 | 61 | 0.01 |
| Nick Thatcher.2015 | 1.46 | 1.15-1.84 | 58 | 0.02 |
| Luis Paz-Ares.2015 | 1.47 | 1.19-1.80 | 52 | 0.04 |
| David R. Spigel 2017 | 1.39 | 1.12-1.72 | 62 | 0.01 |
| Satoshi Watanabe 2019 | 1.26 | 1.12-1.42 | 0 | 0.43 |

Table S1. Sensitivity analysis for objective response rate.
