# Supplementary material for: Anti-EGFR monoclonal antibody plus chemotherapy for treating advanced non-small cell lung cancer: A meta-analysis
Source: Medicine (Baltimore). 2021 Nov 24;100(47):e27954. doi: 10.1097/MD.0000000000027954 (PMC8615333; doi:10.1097/MD.0000000000027954)
Supplement: Supplemental Digital Content [file medi-100-e27954-s008.doc]

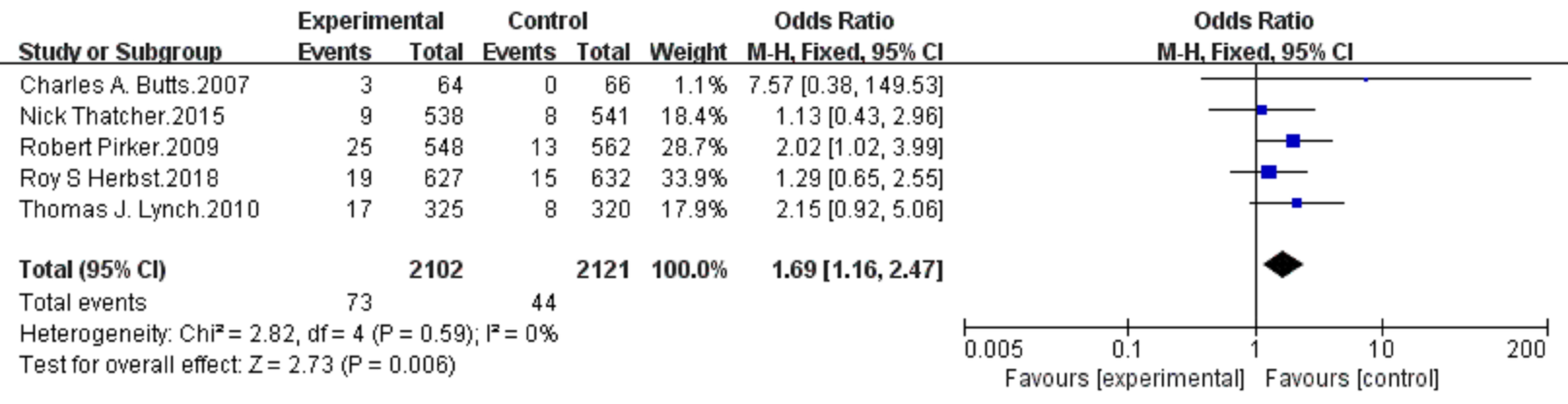


Supplemental Digital Content 13. Forest plots for Diarrhea. Chemotherapy plus anti-EGFR-mAb versus chemotherapy alone for advanced NSCLC. anti-EGFR-mAb= anti-epidermal growth factor receptor monoclonal antibody; NSCLC=non-small-cell lung cancer; CI=confidence interval; Chi^2^=chi-squared test; df=degree of freedom; I^2^=I-squared.





Supplemental Digital Content 14. Forest plots for Dyspnea. Chemotherapy plus anti-EGFR-mAb versus chemotherapy alone for advanced NSCLC. anti-EGFR-mAb= anti-epidermal growth factor receptor monoclonal antibody; NSCLC=non-small-cell lung cancer; CI=confidence interval; Chi^2^=chi-squared test; df=degree of freedom; I^2^=I-squared.





Supplemental Digital Content 15. Forest plots for Infusion-related Reactions. Chemotherapy plus anti-EGFR-mAb versus chemotherapy alone for advanced NSCLC. anti-EGFR-mAb= anti-epidermal growth factor receptor monoclonal antibody; NSCLC=non-small-cell lung cancer; CI=confidence interval; Chi^2^=chi-squared test; df=degree of freedom; I^2^=I-squared.
